# Proceedings of the New York Homœopathic Union

**Published:** 1896-08

**Authors:** Emma D. Wilcox

**Affiliations:** Secretary


### PROCEEDINGS OF THE NEW YORK HOMŒOPATHIC UNION.

The regular meeting of the New York Homœopathic Union was held at 62 West Forty-ninth Street, May 21st, 1896, the President, Dr. Edmund Carleton, in the chair. The following members were present: Drs. Finch, Gillingham, Leverson, O'Brien, Richards, Stanton, Thomson, Young, Wilcox; also Miss Mayer, medical student. Regrets were received from Drs. Fletcher, Howard, and Powel.

Edmund Carleton, M. D., was elected President, and E. D. Wilcox, M. D., Secretary, for the ensuing year.

Reading and discussion of Hahnemann's *Chronic Diseases*, Vol. I, pp. 156 to 169 inclusive (Hempel's translation), was then taken up (p. 133, Tafel's translation).

Question of the accuracy of Tafel's new translation, p. 122, last half of the paragraph, having arisen at the last meeting, the opinion of Dr. Fincke was sought, with the following result:

"BROOKLYN, May 5th, 1896.

"DEAR DR. CARLETON:—The German text is: 'Man wähns ja nicht, dass die Zeit der angegebnen, ungefähren Wirkungs-Dauer kaum abgewartet werden durfe, un eine andre antipsorische Arzner zu geben—*dass man also mit der Abwechslung eilen musse um die Kurzubeschleunigen.*'

"The *literal* translation is: 'One imagines (imperative) on no account that the time of the stated probable duration of action need scarcely be waited for in order to give again another antipsoric medicine—*that, therefore, one must hasten with the alternation in order to accelerate the cure.*'

"'You takes your choice!'

"Yours fraternally,

"B. FINCKE."

There was unanimous concurrence with Hahnemann's statement that "several antipsorics are generally required for the cure of a chronic disease. If the physician alternates his remedies in rapid succession, this is a sure sign that he has not chosen his remedies with strict reference to their homœopathic action, or has but carelessly studied the existing series of symptoms. The homœopathic physician is very apt to commit this mistake in urgent cases of both chronic and acute diseases, especially when the patient is dear to him. The practitioner cannot be sufficiently on his guard against this practice." Individual experiences were mentioned in confirmation of the foregoing, with stress upon the number of remedies and length of time required. Time is an important factor in the treatment of a chronic case.

The succeeding paragraphs, relating to mesmerism and olfaction, elicited the fact that few present had had much experience with either. Olfaction, however, had been successful in the cases cited; and there was great willingness to employ this method of administering medicines when required. Hahnemann's statement that "the remedy acts just as powerfully by communicating its medicinal influence to the system through the nasal fossa and the lungs as if a dose of the remedy had been swallowed" was declared to be reasonable.

Note to page 165 (H.).

"There are hypercritical homœopathic physicians who were afraid that even the sugar of milk might obtain medicinal qualities from being long kept in a bottle, or from long trituration. Long-continued experiments have convinced me that this apprehension is unfounded, etc." Dr. Stanton remarked that the imagination of some nervous patients is so vivid that they get symptoms from innocent placebo. Dr. Finch—"Sugar, Natrum-mur., Lycopodium, must be triturated highly to produce symptoms."

The subject of intercurrent remedies received much attention. Dr. Young deprecated their use, unless the necessity is too great to be ignored with safety. He lets the patient stand the interruption; and believes that he is finally cured sooner by following that course than by interfering to relieve the temporary malady. Dr. Finch related Teste's case, where Sepia was effecting a cure under difficulties. The patient became angry, for some cause. Immediately the action of Sepia was interrupted, and it was impossible to cure after that. Also Lippe's case of chronic affection of the chest, cured by the appropriate remedy. Presently the patient's leg became affected; he would not endure this, and took medicine that entirely relieved the leg. The chest trouble then returned and could not be cured. This paragraph in the *Chronic Diseases* relating to the treatment of intercurrent maladies is to him (Dr. Finch) of more importance than most any other part of the book. It reads thus:

"Among the mishaps which disturb the treatment only in a temporary way I enumerate: Overloading the stomach (this may be remedied by hunger—*i. e.*, by taking a little thin soup instead of the meal and a little coffee); disorder of the stomach from fat meat, especially from eating pork (to be cured by fasting and Pulsatilla); a disorder of the stomach which causes rising from the stomach after eating, and especially nausea and inclination to vomit (by highly potentized Antimonium-crudum); taking cold in the stomach by eating fruit (by smelling of Arsenicum); troubles from spirituous liquors (Nux-vomica); disorder of the stomach with gastric fever, chilliness and cold (Bryonia-alba); fright—when the medicine can be given at once, and especially when the fright causes timidity, by Poppy-juice-opium—but if aid can only be rendered later, or when vexation is joined with the fright, by Aconite; but if sadness is caused by the fright, by Ignatia-seeds; vexation, which causes anger, violence, heat, irritation, by Chamomilla (but if beside the vexation there is chilliness and coldness of the body, by Byronia); vexation with indignation, deep internal mortification (attended with throwing away what was held in the hand) by Staphisagria; indignation with silent internal mortification by Colocynthis; unsuccessful love with quiet grief (by Ignatia); unhappy love with jealousy (by Hyoscyamus); a severe cold (next to keeping the house or the bed), by Nux-vomica; when diarrhœa resulted, by Dulcamara; or if followed by pains, by Coffea-cruda; or if followed by fever and heat, by Aconite; a cold which is followed by suffocative fits (by Ipecacuanha); colds followed by pains and inclination to weep (by Coffea-cruda); cold with consequent coryza and loss of the sense of smell, and of taste (by Pulsatilla); overlifting or strains (sometimes by Arnica, but most certainly by Rhus-toxicodendron); contusions and wounds inflicted by blunt instruments (by Arnica); burning of the skin (by compresses of water mixed with a dilution of highly potentized Arsenicum, or uninterrupted application for hours of alcohol heated by means of very hot water); weakness from loss of fluids and blood (by China); homesickness with redness of the cheeks (by Capsicum)."

The reading of the section relating to intermediate diseases (which are generally fevers) led Dr. Gillingham to give the dictum of Dr. Bayard: "Sulphur is chronic Rhus; and Calc-carb. is chronic Sulphur."

Drs. Finch and Wilcox reported cases of Rhus poisoning successfully treated with high Rhus. Dr. Young had seen Rhus-radicans succeed where Rhus-tox. had failed. This precipitated a long discussion of the alleged identity of the two drugs. Botanists admitted no difference. Dr. Thomson said that the tincture of Rhus-tox. had a shade of color different from that of Rhus-rad. Dr. Carleton was glad that others had been able to cure poison cases with either Rhus-tox. or Rhus-rad. in high potency. He was once persuaded to try that kind of practice, but without success. He never believed in it, but had been persuaded to it by others. He now always selects another drug—often Cantharides. In the *Symptomen Codex* may be found the pathogenesis of Rhus-radicans—"proved and the symptoms arranged by Dr. B. F. Joslin, with the co-operation of Drs. S. B. Barlow, E. Bayard, R. M. Bolles, B. F. Bowers, R. A. Snow, J. Taylor, W. Williamson, and C. Wright. A few symptoms are from Drs. Bute and Horsfield." A note at the end of the article argues the difference between the two drugs. This sentence appears in it: "Moreover, in these provings the radicans has evinced great power and several properties not hitherto discovered in the Toxicodendron." Subsequent compilations have combined all the symptoms, and published them under Toxicodendron against the wishes of the provers of radicans.

Meeting adjourned.

EMMA D. WILCOX, *Secretary.*

